# Authors' opinions on publication in relation to annual performance assessment

**DOI:** 10.1186/1472-6920-10-21

**Published:** 2010-03-09

**Authors:** Robin L Walker, Lindsay Sykes, Brenda R Hemmelgarn, Hude Quan

**Affiliations:** 1 Department of Community Health Sciences, University of Calgary, Calgary, Canada; 2 Department of Medicine, University of Calgary, Calgary, Canada

## Abstract

**Background:**

In the past 50 years there has been a substantial increase in the volume of published research and in the number of authors per scientific publication. There is also significant pressure exerted on researchers to produce publications. Thus, the purpose of this study was to survey corresponding authors in published medical journals to determine their opinion on publication impact in relation to performance review and promotion.

**Methods:**

Cross-sectional survey of corresponding authors of original research articles published in June 2007 among 72 medical journals. Measurement outcomes included the number of publications, number of authors, authorship order and journal impact factor in relation to performance review and promotion.

**Results:**

Of 687 surveys, 478 were analyzed (response rate 69.6%). Corresponding authors self-reported that number of publications (78.7%), journal impact factor (67.8%) and being the first author (75.9%) were most influential for their annual performance review and assessment. Only 17.6% of authors reported that the number of authors on a manuscript was important criteria for performance review and assessment. A higher percentage of Asian authors reported that the number of authors was key to performance review and promotion (41.4% versus 7.8 to 22.2%). compared to authors from other countries.

**Conclusions:**

The number of publications, authorship order and journal impact factor were important factors for performance reviews and promotion at academic and non-academic institutes. The number of authors was not identified as important criteria. These factors may be contributing to the increase in the number of authors per publication.

## Background

In the past fifty years there has been a substantial increase in the volume of published research. The number of papers cited by Pub Med each year has also grown linearly, to a current total exceeding 18 million [1]. The increase in publications has been accompanied by an increase in the number of authors per scientific publication [2-5]. In fact the average number of authors in major medical journals has increased from 4.5 in 1980 to 6.9 in 2000, and single authored articles have diminished [5,6]. For example, in January 2009 a manuscript was published with almost 3000 authors [7,8]. This increase has resulted in carefully defined criteria to be established by journal editors to define authorship requirements, with limitations on the number of authors established by some medical journals [3,5].

Researchers have implicated the increased complexity of research (including technological advances) and the increase in multi-center and multidisciplinary research collaborations to the growth in the number of authors per manuscript. However, it can be argued that other factors have a stronger impact on this so-called "author inflation". These factors include professional and social advancement (promotion), tenure, prestige, funding, and honorary authorships. Promotion and tenure committees use different procedures, criterion, and weight to evaluate researchers within faculties and across institutions; both academic and non-academic. Some institutions may reward publications, particularly for publication in "high impact journals" [9]. Therefore, there is significant pressure exerted on researchers to produce publications in top journals [3]. The journal impact factor is frequently used as a proxy for the importance of a journal in its particular field [10].

We surveyed corresponding authors in published medical journals to determine their opinion on aspects of publication (authorship order, number and journal impact factor) in relation to performance review and promotion.

## Methods

### Identifying Journals, Annals and Articles

We identified potential authors to survey through the following steps. A listing of medical journal names, classified by medical field, were initially extracted from the Journal Citation Report (JCR) of 2006 [11], which were classified according to medical field. The JCR offers a systematic, objective mean to critically evaluate journals in over 7600 scholarly and technical journals, from more than 3300 publishers in over 60 countries. Specifically we extracted journal names that were indexed as 'medicine general and internal' and medically related annals. From that list we included all articles published in June 2007 (month chosen for article extraction) which were online at the time of the literature search in August 2007 (see Appendix 1). Articles were then selected if they were published in English in June 2007, included corresponding author information, and were original research studies.

### Data Collection

The following data was extracted from each article identified: research design (randomized controlled trial (RCT) or non-RCT), research field (bio-medical or non-biomedical), number of authors listed, position of corresponding author on manuscript (first, last, middle), as well as corresponding author's name, country of residence and email address.

Each corresponding author was sent a survey via email. After three weeks a reminder email was sent to non-respondents, with a final reminder email sent to non-respondents two months later. The survey included questions regarding authorship order, decision of authorship position on the manuscript (first, last, middle author) and author affiliation (position at institution). The survey also included questions regarding whether the number of publications, number of authors, authorship order and journal impact factor contributed towards their performance review and promotion at their institution.

### Statistical Analysis

Surveys with missing responses were excluded from the analysis. Statistical analyses were performed using Stata Verson 10 (Stata, College Station TX). To address the purpose of this study the data analysis was primarily descriptive.

The study was approved by the Conjoint Health Research Ethics Board, University of Calgary.

## Results

**Figure 1:**
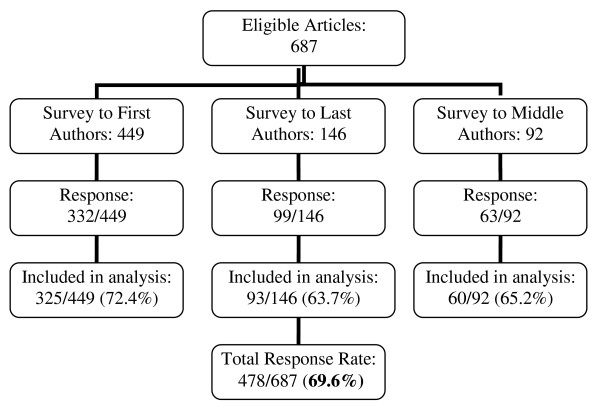
**Flow chart of eligible articles to study response rate**.

We identified 687 original research articles published in June 2007 from 72 journals which met our eligibility criteria. Of 687 surveys, 494 (71.9%) were returned. After excluding those with missing data (n = 16), 478 surveys were available for the analysis, for a final response rate of 69.6% (Figure 1). The response rate was slightly higher for first authors (72.4%) compared to last (63.7%) and middle authors (65.2%).

The characteristics of articles and corresponding authors are shown in Table 1. The majority of the research articles were non randomized controlled trials (93.5%), with 56.7% of articles bio-medical or laboratory based studies. Eighty-percent of articles were published in a journal with an impact factor less than 6. The average number of authors listed per manuscript was 6; five articles had one author listed. Among articles with a journal impact factor of ≥ 10, 60% (n = 18) had more than 10 authors listed on the manuscript. The majority of corresponding authors were from Europe (38.7%), North America (35.0%), and Asia (20.7%) and held an academic position within their institution (68.8%).

**Table 1:** Characteristics of Articles and Corresponding Authors

	n (%)
**ARTICLES**	
**Research field and type**	
Bio-medical/Lab	271 (56.7)
Non Bio-medical	207 (43.3)
	
RCT*	36 (7.5)
Non-RCT*	442 (92.5)
	
**Journal Impact Factor**	
≤ 1	178 (38.2)
2-5.9	200 (41.8)
6-9.9	70 (14.6)
≥ 10	30 (6.3)
	
**Number of Authors**	
1	5 (1.1)
2-4	153 (32.0)
5-9	256 (53.6)
10+	64 (13.4)
**CORRESPONDING AUTHORS**	
	
**Country**	
North America	167 (35.0)
Europe	185 (38.7)
Asia	99 (20.7)
Australia/New Zealand	14 (2.9)
South America	8 (1.7)
Other	5 (1.0)
	
**Position at Institution**	
Academic	329 (68.8)
Trainee	75 (15.7)
Non Academic	74 (15.5)

* RCT = randomized controlled trial

Authors self-reported that the number of publications (78.7%), the journal impact factor (67.8%) and being the first author (75.9%) were most influential for their annual performance review and assessment (Table 2). Only 17.6% of authors reported that the number of authors on a manuscript was an important criterion for performance review and assessment. Even when we stratified the variables by corresponding author characteristics, the results remained similar (Table 3). Compared to other authors a higher percentage of Asian authors reported that the number of authors was key to performance review and promotion (41.4% versus 7.8% to 22.2%).

**Table 2:** Corresponding Author's Opinions on Publication Impact on Annual Performance Review and Promotion

	n (%)
**Number of Publications**	
Yes	376 (78.7)
No	102 (21.3)
	
**Number of Authors**	
Yes	84 (17.6)
No	394 (82.4)
	
**Journal Impact Factor**	
Yes	324 (67.8)
No	154 (32.2)
	
Author Order*	
First	363 (75.9)
Last	32 (6.7)
Corresponding	44 (9.2)
Other	39 (8.2)

*The question used was: "Which authorship order is of greatest merit in relation to performance review and promotion?"

**Table 3:** Corresponding Author's Opinions on the Relevance of Publication Impact to Performance Review and Promotion Stratified by Corresponding Author's Characteristics

	# Publications		# Authors		Journal Impact Factor	
	n (%)	P-value	n (%)	P-value	n (%)	P-value
**Corresponding**						
**Author**						
First Author	248 (76.3)		48 (14.8)		210 (64.8)	
Other	128 (83.7)	>0.05	36 (23.5)	<0.05	113 (73.9)	<0.05
**Position at Institution**						
Academic	285 (86.6)		69 (21.0)		234 (71.3)	
Trainee	49 (65.3)		9 (12.0)		39 (52.0)	
Non-academic	42 (6.8)	<0.001	6 (8.1)	<0.05	50 (67.6)	<0.01
**Research Field**						
Bio-medical/Lab	206 (76.0)		58 (21.4)		178 (65.9)	
Non Bio-medical	169 (82.0)	>0.05	26 (12.6)	>0.05	144 (69.9)	>0.05
**Country**						
North America	139 (83.2)		13 (7.8)		103 (62.0)	
Europe	133 (71.9)		24 (13.0)		129 (69.7)	
Asia	83 (83.8)		41 (41.4)		72 (72.7)	
Other	21 (77.8)	<0.05	6 (22.2)	<0.001	19 (70.4)	>0.05

Corresponding authors were asked when the initial decision was made regarding the authorship order for the manuscript. Fifty eight percent reported it was decided prior to writing the manuscript, 28.9% during writing of the manuscript, and 12.0% after the manuscript was written. Only 12.0% of authors reported that the authorship order changed after the initial authorship order decision was made.

Of the surveys, 26.4% of authors believed that the number of authors on a manuscript should be limited by a journal. Among those authors, the average limit they suggested was 6.

## Discussion

We conducted a survey among corresponding authors to assess their opinions of publication impact in relation to performance review and promotion. Overall we found that most respondents considered the journal impact factor, number of publications, and authorship order important to their performance review and promotion. The number of authors on a paper did not seem to affect performance review and promotion except among Asian authors.

In general, performance reviews are challenging. Promotion and tenure committees attempt to include objective criteria, yet most use varying procedures, criterion, and weighting schemes to evaluate researchers for promotion and tenure [7]. The absolute number of publications and authorship order are only a few variables that institutions may use in performance review and promotion. Some institutions also evaluate the journal, enhancing the desire for researchers to publish in top journals. Our study results show that the journal impact factor is important to author's performance review and promotion. This was true regardless of the corresponding author's position at their institution or on the manuscript, and country of residence. Interestingly even non-academic authors reported that it was an important aspect of their evaluation.

There is pressure on researchers to publish due to evaluation procedures, and the desire for prestige, employment and tenure [12]. Coinciding with this pressure, we found that the number of publications is important to performance review and promotion. Could this pressure be responsible for the large number of manuscripts being published? It has been suggested that this pressure may contribute to authorship misconduct, such as "undeserved authorship" and "honorary authorship" [3,4,12]. This has resulted in some journal editors requiring a written rationale regarding authorship and a published explanation of each author's contribution [13].

Journal editors have also discussed the possibility of limiting the number of authors on a manuscript to try and reduce authorship misconduct [14]. Guidelines produced by the International Committee of Medical Journal Editors and the American Psychological Association have suggested that authorship should be limited to those who have substantially contributed to the manuscript [7,15]. In our study only a small proportion of authors felt that medical journals should limit the number of authors on published manuscripts. Perhaps this is because researchers want to take advantage of every opportunity to publish due to the publication pressure placed on them by institutions. Other reasons may include researchers wanting to include senior authors on manuscripts to give them more "power" and creditability [16]. Additionally, there has been an increase in group authorship and researchers may want their names to be listed on manuscripts as many institutions do not recognize these as individual research contributions in performance review and promotion [5].

There are few organizations [15] with specific authorship guidelines regarding author order and a suggested time period for when this should be decided. In our study we found that the majority of authors established authorship order prior to writing the manuscript. Although some journals require authorship to be alphabetical, the Journal of American Statistical Association suggests that authorship order should be determined by the magnitude of intellectual contribution to the project, rather than by status, seniority or power within the group [7].

Some universities only merit first or corresponding authors in annual performance review and promotion. Other universities recognized second or third authors; however being first or corresponding author has the most recognition and financial compensation [17]. In fact some Asian countries, such as China and Japan, give incentives for first and corresponding authors and for publications in high impact factors journals. However, this type of recognition may limit research collaboration. This is consistent with the results from our study in which only Asian authors reported that the number of authors on a manuscript was important to their performance review and promotion whereas in other continents this was not as significant.

Our study has limitations. First, we only surveyed corresponding authors and not those responsible for implementing performance reviews and promotion (for example department heads). The authors surveyed however should be aware and able to accurately report the criteria used by their institutions for performance reviews and promotion. Second, we surveyed authors from medical-related journals that were published in English, thus our finding may not be generalizeable to other disciples such as mathematics and physics. In addition we only surveyed authors with published articles, thus we do not know the opinions of non-published authors. Finally, there was a 30.4% non-response rate; we have no way of assessing if the opinions of these authors are consistent with those of the respondents.

## Conclusion

In summary we found that corresponding authors published in English language medical journals considered the number of publications, authorship order and journal impact factor to be important factors for their performance reviews and promotion. The number of authors on a publication was not identified as important criteria. These factors may be contributing to the increase in the number of authors per publication, and the so-called author inflation.

## Competing interests

The authors declare that they have no competing interests.

## Authors' contributions

RW participated in the conception and design, analysis and interpretation of data, drafting the manuscript, and statistical analysis. LS participated in the conception and design, acquisition of data, drafting of the manuscript, and administrative and technical support. BH participated in the conception and design, analysis and interpretation of data, critical revision of the manuscript for important intellectual content, and supervision. HQ participated in the conception and design, analysis and interpretation of data, critical revision of the manuscript for important intellectual content, and supervision. All authors read and approved the final manuscript.

## Appendix 1: Journal Included in the Survey

Am Fam Physician

Am J Chinese Med

Am J Manag Care

Am J Med

Am J Med Sci

Am J Prev Med

Arch Intern Med

Aviat Space Envir Md

Brit Med J

Can Fam Physician

Can Med Assoc J

Chinese Med J-Peking

Croat Med J

Curr Med Res Opin

Fam Med

Fam Pract

Hosp Med

Indian J Med Res

Intern Med J

Internal Med

Irish J Med Sci

Israel Med Assoc J

J Eval Clin Pract

J Korean Med Sci

J Lab Clin Med

J Natl Med Assoc

J Pain Symptom Manag

J R Soc Med

J Travel Med

J Womens Health

J Am Med Assoc

Journal of Cutaneous Pathology

Mayo Clin Proc

Med J Australia

Med Klin

Med Prin Pract

Mil Med

Neth J Med

New Engl J M

Pain Med

Plos Med

Postgrad Med J

Qjm-Int J Med

Rev Med Chile

Rev Med Interne

Saudi Med J

South Med J

Swiss Med Wkly

Tohoku J Exp Med

Transl Res

Upsala J Med Sci

Wien Klin Wochenschr

Yonsei Med J

Ann Acad Med Singap

Ann Allerg Asthma Im

Ann Bot-London

Ann Emerg Med

Ann Endocrinol Paris

Ann Epidemiol

Ann Fam Med

Ann Hematol

Ann Intern Med

Ann Med

Ann Neurol

Ann Oncol

Ann Pathol

Ann Plas Surg

Ann Rheum Dis

Ann Surg

Ann Surg Oncol

Ann Thorac Surg

Ann Trop Paediatr

## Pre-publication history

The pre-publication history for this paper can be accessed here:

http://www.biomedcentral.com/1472-6920/10/21/prepub
